# Kidney stones and oxidative stress. Types of papillary renal calculi

**DOI:** 10.1007/s00240-025-01746-9

**Published:** 2025-05-13

**Authors:** F. Grases, A. Costa-Bauzá

**Affiliations:** Renal Lithiasis and Pathological Calcification Group, Research Institute of Health Sciences (IUNICS-IdISBa), University of Balearic Islands, Palma de Mallorca, Spain

**Keywords:** Renal papillary stone, Calcium oxalate, Randall’s plaque, Oxidative stress, Renal tubular plugs

## Abstract

Reactive oxygen species can promote the formation of kidney stones, and this process requires the participation of cells associated with the renal papilla. Here, we present a revised interpretation of the characteristics of the different types of renal papillary stones and the possible pathways responsible for their formation. We examined kidney stones from a biobank that contains 15,000 stones and identified five different types of papillary stones. Type I stones are calcium oxalate monohydrate (COM) stones that clearly have Randall’s plaque but have no renal tubules near the stone-tissue junction. Type II stones are COM stones that have Randall’s plaque and calcified renal tubules around the stone-tissue junction. Type III stones are calcium oxalate dihydrate (COD) stones that have a stone-tissue junction and calcified renal tubules. Type IV stones are COM stones containing important deposits of uric acid and/or Na or K urates that occur around stone-tissue junction, together with apatite phosphate, and may also contain bacterial imprints. Type V stones are small COM calculi that have no hydroxyapatite deposits at the stone-tissue junction. Oxidative stress of papillary tissues can generate heterogeneous nucleants that promote the crystallization of calcium phosphate and calcium oxalate, and urine composition determines the type of papillary stone ultimately develops. An active immune response can limit or prevent the development of these stones by eliminating the intra-tissue hydroxyapatite deposits or promoting the regeneration of the outer uroepithelium.

## Introduction

Oxidative stress can lead to the formation of renal stones, but is not responsible for all renal stones. Stones that form in response to reactive oxygen species (ROS) require the participation of renal tissues, especially the renal papilla [1, 2]. Although stones induced by ROS have a papillary origin, papillary stones are diverse and form by different underlying pathways. It is important to identify these different pathways so that effective prevention measures can be used. In all cases, the cause of oxidative stress and its consequences must be considered. In this regard, it is necessary to consider that the development of crystals in the upper urinary tract is a consequence of the high supersaturation of substances that crystallize and/or the presence of heterogeneous nucleants that induce stone formation. In fact, two clearly different general pathways can be considered.

The first pathway occurs when there are normal levels of urinary supersaturation in calcium oxalate (as in healthy individuals), and the ROS induce tissue lesions. This leads to an immune response (accumulation of macrophages and inflammatory molecules) and the production of various types of cellular debris which can act as heterogeneous nucleants that induce crystal formation. If the lesion occurs within the papillary tissue and is irrigated by interstitial fluid with a pH of 7.4 (plasma pH), then apatite phosphate deposits form. If these deposits are not eliminated by the immune system, they may grow, cross the cuboidal epithelium that covers the papilla and, upon contact with urine, induce the growth of calcium oxalate on the deposit [1, 2].

The second pathway occurs when there is a high concentration of calcium in the urine (possible hypercalciuria) and a urinary pH above 6.2 in the renal tubules, conditions that generate significant crystals inside the tubules. When these crystals contact the cells of the tubules, they can induce lesions. The resulting calcium oxalate crystals can alter membrane structure and increase the level of ROS by adherence to tubular cell membrane, leading to crystal internalization. These same processes also function in the induction of cell death [3]. These lesions also activate the immune system, which then generates inflammatory cells and molecules that induce the development of more calcium oxalate crystals. This is followed by obstruction of the renal tubules, and generation of plugs at the tip of the papilla. When these plugs encounter urine, papillary stones can form [4, 5].

Although each of these general pathways can lead to the formation of papillary stones, the underlying causes are different and they therefore require different methods for therapeutic prevention. In this paper, we present a revision of the different types of papillary stones and the possible pathways responsible for their formation.

## Materials and methods

The renal papillary stones described herein are part of the collection of 15,000 kidney stones that are at the Renal Lithiasis Research Laboratory of the IUNICS of the University of the Balearic Islands and the Kidney Stone Biobank (BICUIB).

The methodology used to examine kidney stones was described previously [6]. The general scheme consisted of initial observations using stereoscopic microscopy (MOTIC SMZ-161, MoticEurope, Barcelona, Spain). For stones of probable papillary origin, the surface of the stone was examined to identify the presence of calcium oxalate monohydrate (COM), calcium oxalate dihydrate (COD), or uric acid. The area of a possible union of the stone with the papilla was also determined; this area was studied in detail, and the presence of phosphate-apatite, renal tubules and other crystalline deposits was also investigated. To confirm the nature of these deposits, samples were also examined by scanning electron microscopy (Hitachi TM4000 Plus II Desktop Scanning Electron Microscope, Hitachi, Tokyo, Japan) coupled with energy dispersive X-ray microanalysis (Quantax 75 EDS Microanalyzer, Bruker, Berlin, Germany). After this initial analysis each whole stone, the renal calculus was divided in half along a plane that passed through the central area of attachment to the papilla. This allowed for a clear identification of the possible presence of calcified renal tubules and their location relative to apatite phosphate deposits, the location of the apatite relative to development of the stone, and the presence of urates in the area of attachment. The mapping of elements in this area (Ca, P, N, and C) by X-ray microanalysis allowed establishment of the location of different types of deposits within a stone.

## Results

**Fig. 1:**
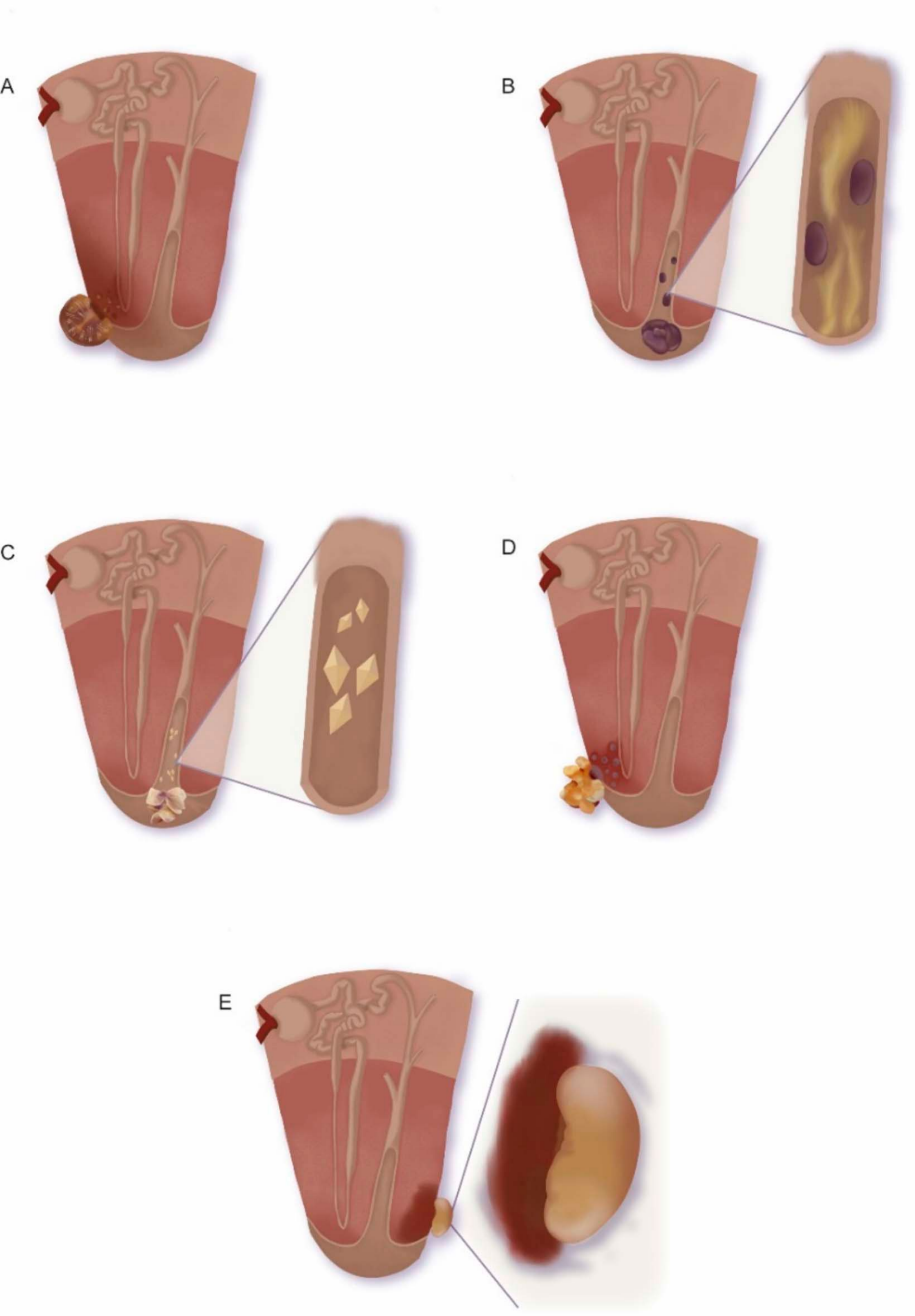
Scheme of development of the different types of papillary renal calculi. (**A**) type I; (**B**) type II; (**C**) type III; (**D**) type IV and (**E**) type V

Our study of a large biobank of kidney stones led to the identification of five different types of renal papillary calculi (Fig. 1). We present representative images of these five types of stones below.

**Fig. 2:**
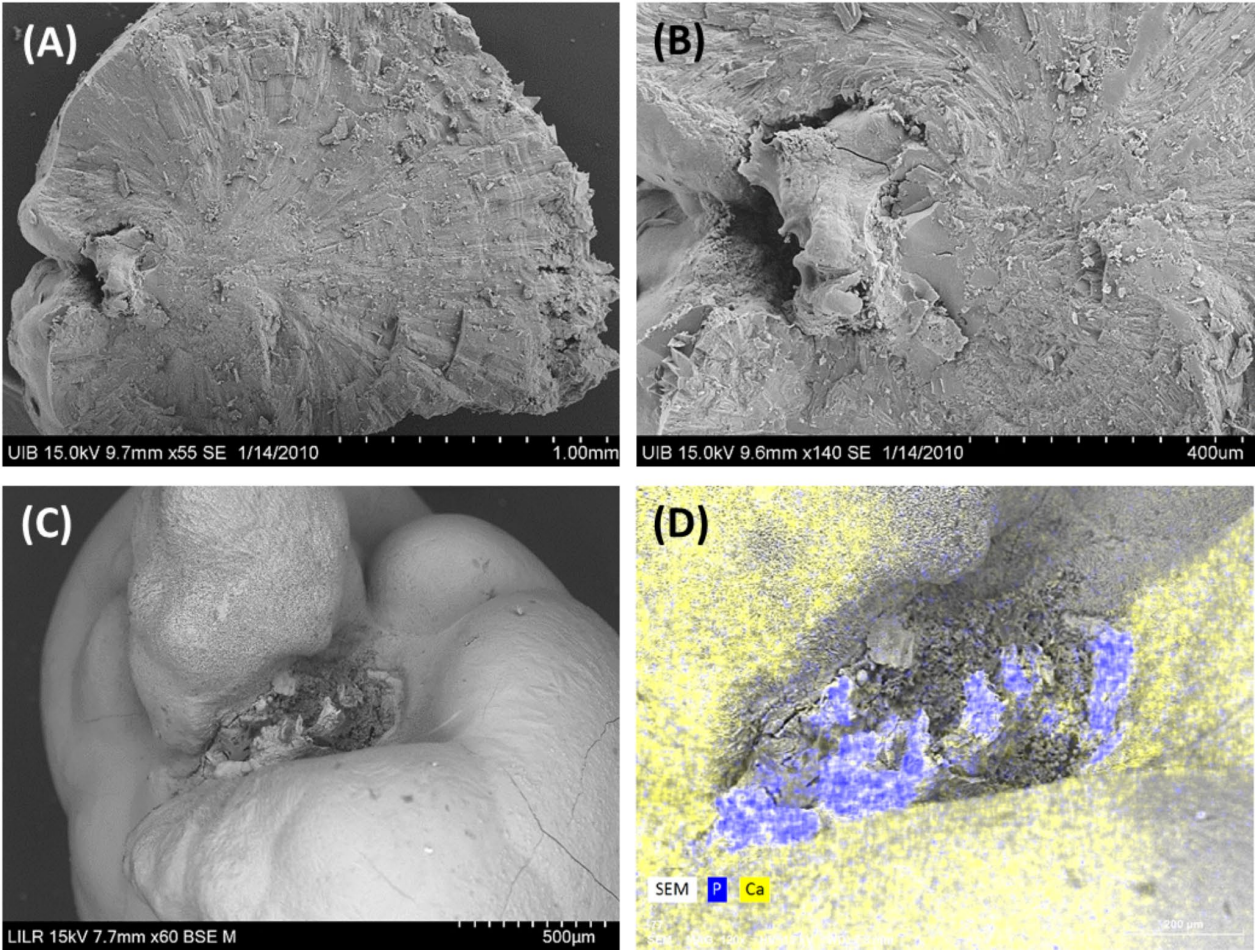
Scanning electron microscopy of two Type I stones. (**A**) Section of a COM papillary stone in which Randall’s plaque was clearly present. (**B**) Detail of Randall’s plaque from A, showing that tubules were undetectable. (**C**) External view of the junction of the papilla of a different COM papillary stone, in which renal tubules were also undetectable. (**D**) Elemental distribution map of the stone from C, obtained by energy dispersive X-ray microanalysis, showing that phosphorous was only located in the region of the stone-tissue junction (Ca: yellow, P: blue)

Type I stones are COM papillary calculi in which the Randall’s plaque (apatite phosphate) is clearly present at the junction with the papilla, but renal tubules were not detectable in this entire area (Fig. 2).

**Fig. 3:**
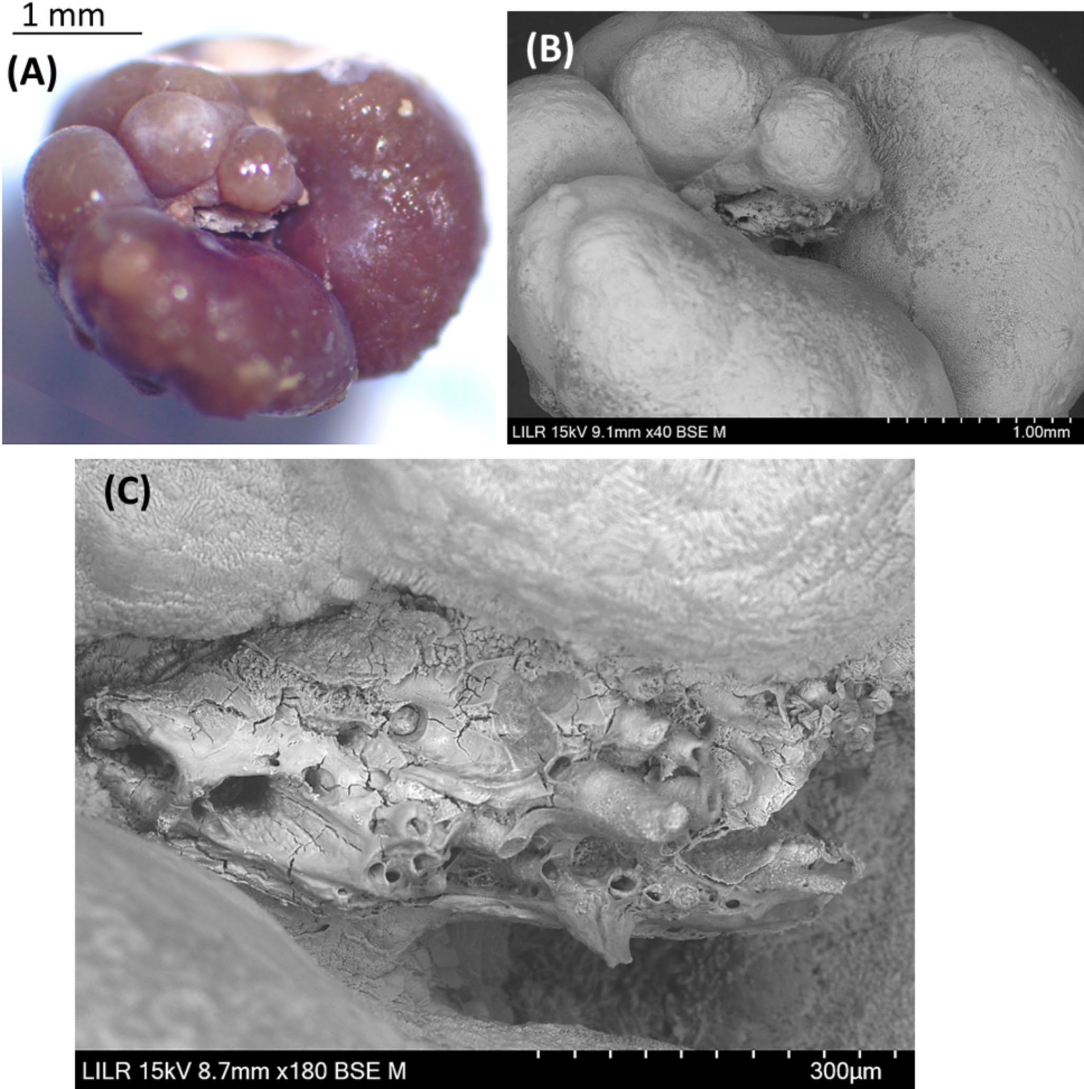
Stereoscopic microscopy (**A**) and scanning electron microscopy (**B**,** C**) of a Type II stone. (**A**,** B**) Whole COM papillary renal stone at the stone-tissue junction, in which calcified renal tubules were present. (**C**) Detail of the calcified renal tubules and plugs

Type II stones are COM papillary calculi in which apatite phosphate is present at the stone-tissue junction (Randall’s plaque) and there are calcified renal tubules (obstructed or unobstructed) and plugs in that area (Fig. 3).

**Fig. 4:**
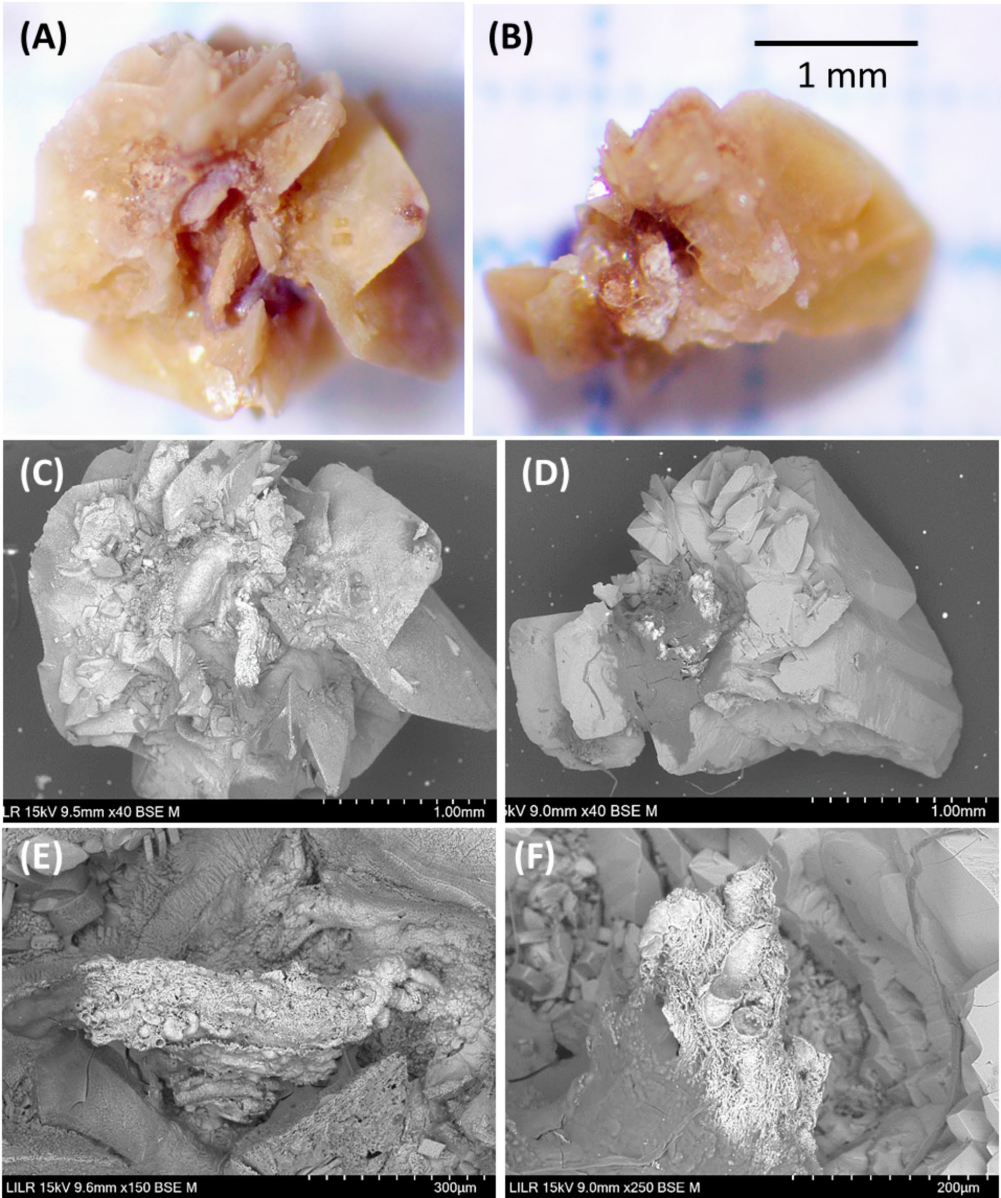
Stereoscopic microscopy (**A**,** B**) and scanning electron microscopy (**C**–**F**) of two Type III stones (one in **A**,** C**, and **E**, and another one in (**B**,** D**, and **F**). Each COD stone had a clear stone-tissue junction (**A**–**D**) and the magnified views show calcified renal tubules (**E**, **F**)

Type III stones are COD stones in which the stone-papilla junction is clearly present, and there are also calcified renal tubules and plugs (Fig. 4).

**Fig. 5:**
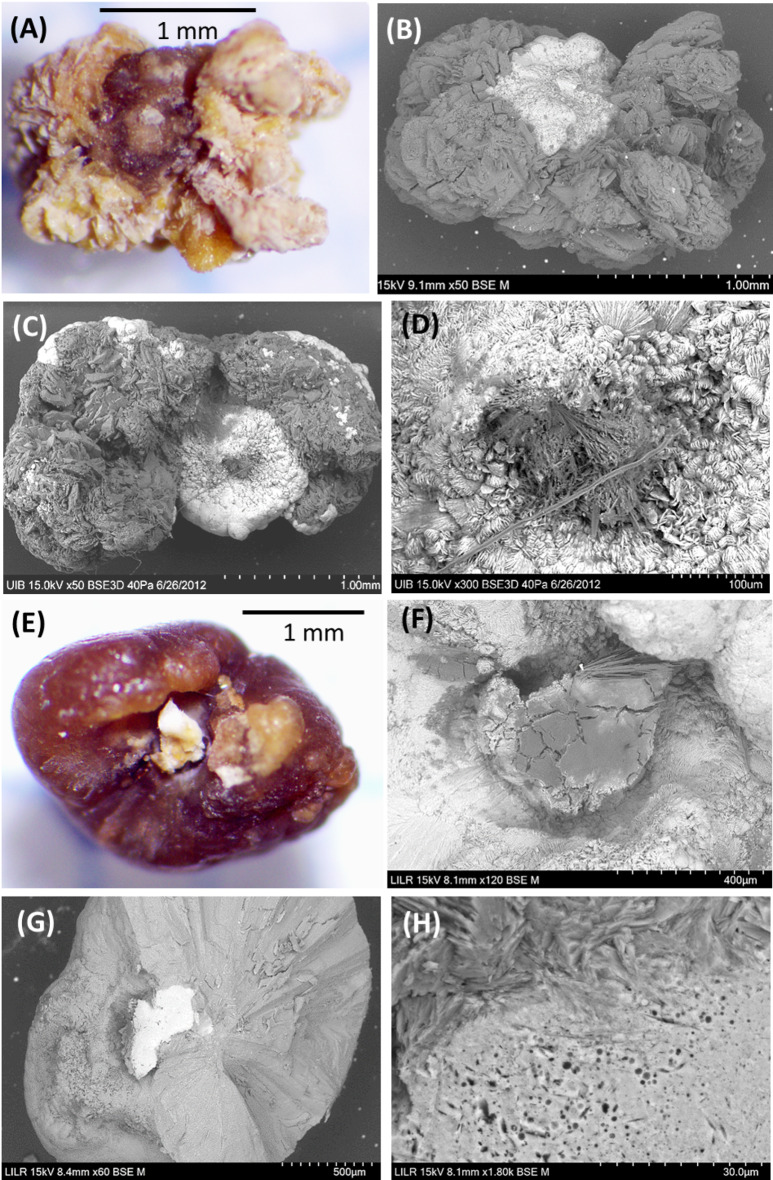
Stereoscopic microscopy (**A**,** E**) and scanning electron microscopy (**B**–**D**, **F**–**H**) of three type IV stones. (**A**) COM papillary stone on which uric acid crystals grew, with the brown area corresponding to COM and the yellow-orange area corresponding to uric acid. (**B**) SEM of the stone in A, in which the white area (heavier elements) indicates COM and the gray area (lighter elements) indicates uric acid. (**C**) A second COM papillary stone containing sodium urate needle-like crystals in the area of the stone-tissue junction. (**D**) Detail of the stone in C, showing sodium urate crystals (gray area). (**E**) A third COM papillary stone that contained hydroxyapatite at the stone-tissue junction. (**F**) SEM of the stone in E shows sodium urate crystals in the junction area. (**G**) Section of the renal calculus from E and F, showing a large white area (hydroxyapatite) at the stone-tissue junction. (**H**) Detail of the hydroxyapatite in G, showing bacterial imprints

Type IV stones are COM papillary stones that contain uric acid or urates (Na or K). The COM crystals develop on the papilla and uric acid then forms on these crystals. In some cases, urates are also present near the junction with the papilla, together with apatite phosphate, and there may also be bacterial imprints (Fig. 5).

**Fig. 6:**
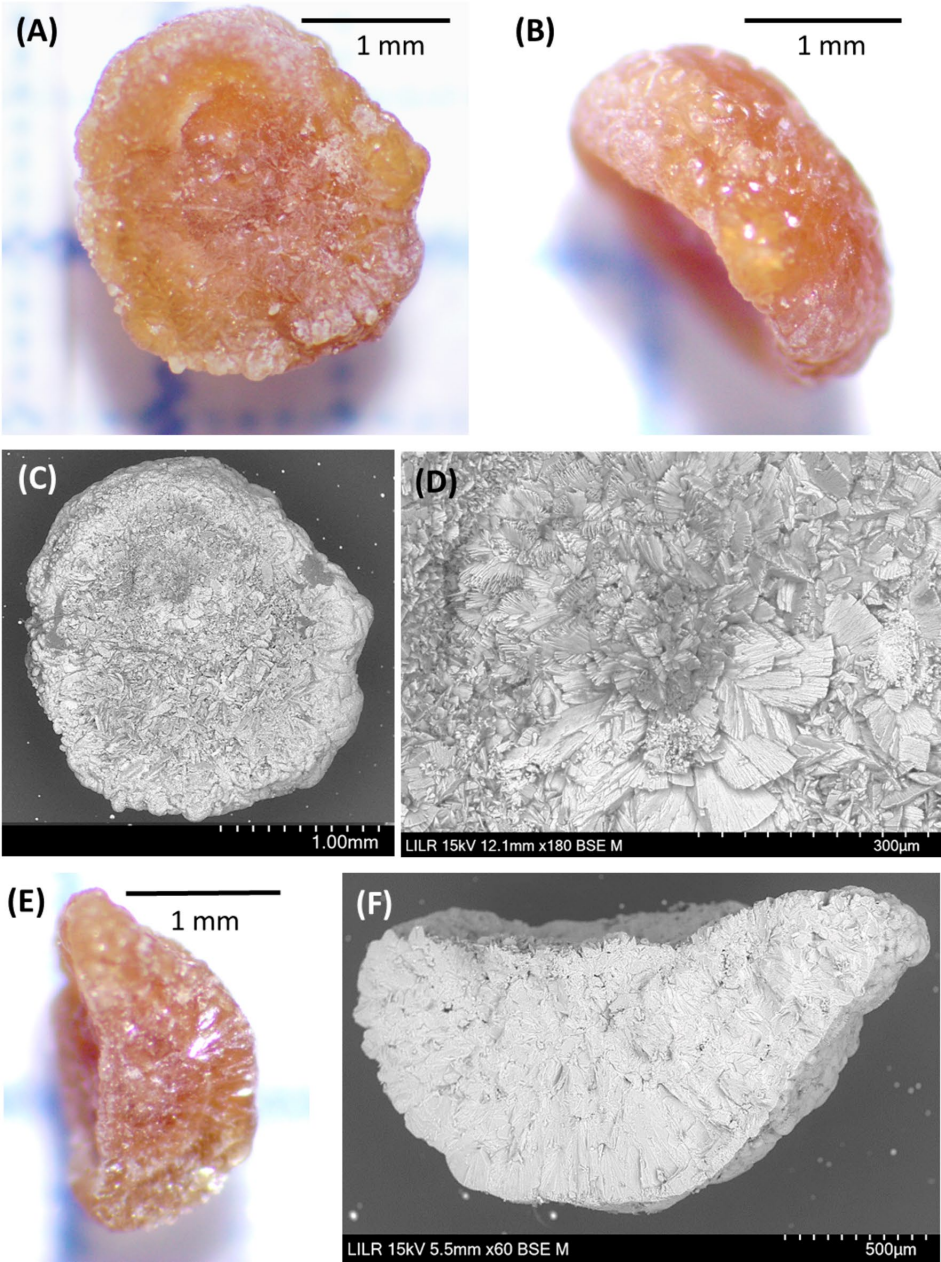
Stereoscopic microscopy (**A**,** B**,** E**) and scanning electron microscopy (**C**, **D**, **F**) of a type V stone. (**A**–**D**) Whole COM papillary stone that had no evidence of hydroxyapatite at the stone-tissue junction. (**E**, **F**) Section of the stone

Type V stones are COM papillary calculi in which there are no hydroxyapatite/apatite phosphate deposits at the stone-tissue junction. These are usually very small stones, and the COM crystals appear to form a columnar structure directly on the papillary tissue or organic matter (Fig. 6).

## Discussion

The typical papillary COM kidney stone is small and generally has a growing surface that is convex and smooth and a concave surface on the opposite side (Fig. 1). In Type I stones (Fig. 2), the concave part is in contact with the kidney tissue and has hydroxyapatite deposits (Randall’s plaque). In fact, these deposits are usually generated inside the papillary tissue, in the thin-loop basement membranes or near the vasa recta [7], both of which are collagen-rich regions [8]. The formation of Randall’s plaque is similar to the mineralization of extracellular matrix at other body locations and implies the presence of cytotoxic substances that generate ROS [9–11]. Oxidation of collagen fibers leads to the production of carboxylate groups that function as heterogeneous nucleants of hydroxyapatite [8]. In fact, collagen fibers in the vicinity of Randall’s plaque are common. Oxidative stress is caused by the excessive generation of free radicals and/or failure of the antioxidant defense mechanism to quench free radicals.

Papillary COM lithiasis is more common in individuals who have professions that involve the inhalation of certain pesticides or organic solvents and in those who consume certain drugs [12]. Moreover, the consumption of cytotoxic products, such as ethylene glycol, and excessive use of certain analgesics can also induce papillary calcifications [13–14]. Interstitial fluid, such as plasma, is supersaturated with calcium phosphates, and calcification can occur if an immunodeficient individual lacks calcification inhibitors [15–19]. This process begins when lesions developing in the papillary tissue generate organic detritus (i.e., oxidized collagen fibers). Due to the high supersaturation of apatitic phosphates in interstitial fluid, a deficiency of low-molecular-weight inhibitors (pyrophosphate, magnesium, phytate derivatives, and citrate [20–21]) causes this organic detritus to attract small calcium-phosphate particles (Posner clusters). This is followed by the growth of these particles [22] and the generation of Randall’s plaque. Some carboxyproteins, such as osteopontin, can bind hydroxyapatite and recruit macrophages that remove these calcifications or prevent their growth [23]. Not all papillary calcifications generate Randall’s plaque and papillary stones, because direct contact between Randall’s plaque and urine is necessary, and some intrapapillary calcifications never contact urine. A previous study showed that the calcium content of the papillae of individuals with papillary stones was higher than that of the papillae of healthy individuals; however, for individuals with unilateral papillary stones, the calcium content is the same in the papillae of both kidneys [24].

**Fig. 7:**
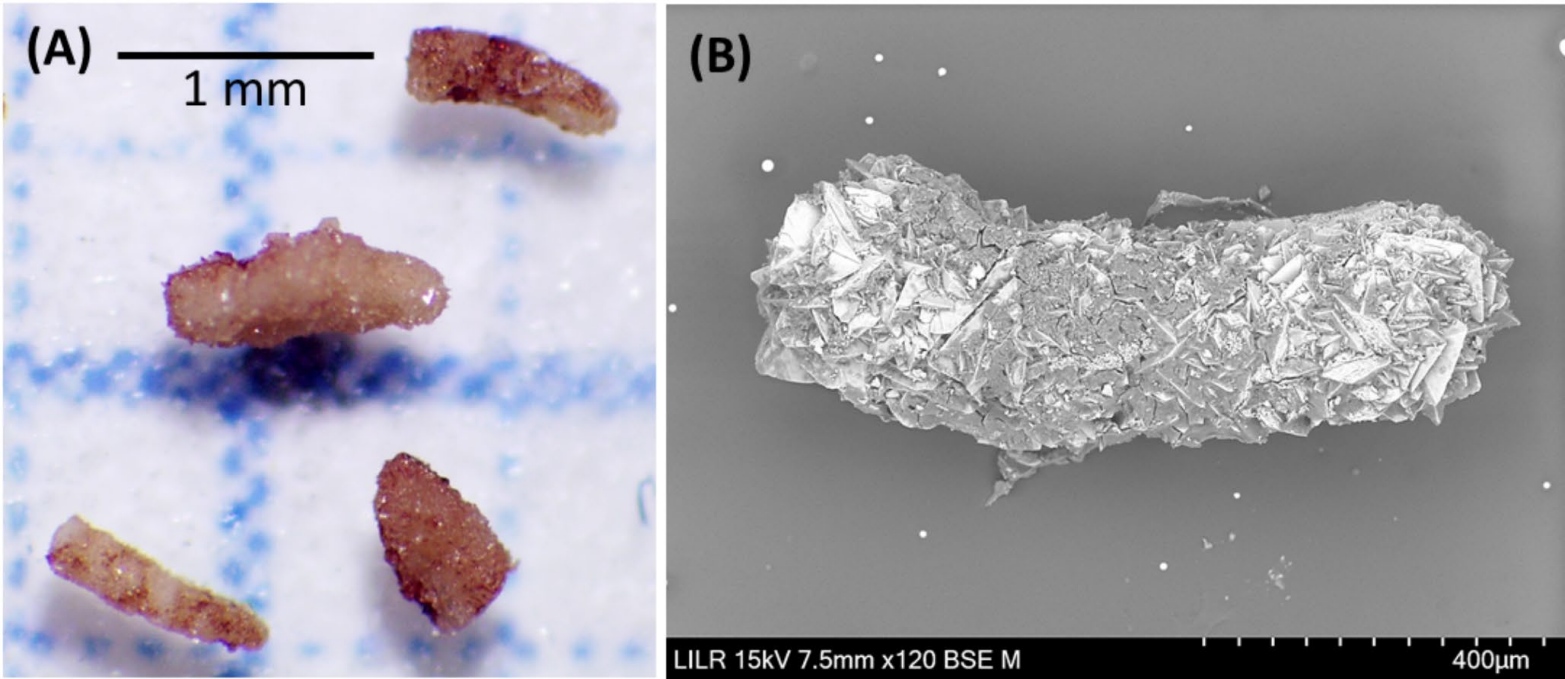
Stereoscopic microscopy (**A**) and scanning electron microscopy (**B**) of Randall’s plug (with blockage of Bellini’s duct) in Types II and III stones collected by percutaneous nephrolithotomy

Patients with Type II and Type III papillary stones have high urinary concentrations of calcium and/or oxalate due to the high supersaturation of calcium oxalate. This can lead to the formation of COM or COD crystals in the renal tubules, which can then induce lesions in the tubular epithelium. Tubular plugs form as a consequence of this whole process (Fig. 7). A urinary pH above 6.2 favors plug formation. When these structures and the damaged tubules reach the tip of papilla and contact urine, the papillary COM stones can develop at the tip of the papilla. The generation of these lesions can lead to the formation of Randall’s plaques. In these stones, the presence of calcified tubules around the junction with the papilla is evident (Figs. 3 and 4). The morphology of these stones can vary: a stone can consist of COD crystals with small amounts of hydroxyapatite and fragments of calcified renal tubules in the area of the stone-tissue junction (Fig. 4); alternatively, a typical papillary COM stone may appear that has COD crystals in the outer convex surface, with calcified renal tubules and Randall’s plaque in the area of the stone-tissue junction (Fig. 3). The presence of plugs in the collecting ducts affects many more nephrons than Randall’s plaque, so these lesions more commonly lead to the loss of renal function [25]. COD papillary calculi are difficult to identify because due to their irregular morphology, the area where they join the papilla often goes unnoticed.

When urine has high concentrations of uric acid and a pH below 5.5, small papillary COM stones can occasionally form, and these stones are covered by a significant layer of uric acid crystals (Type IV stones, Fig. 5). It is evident that uric acid has been generated on the COM that constituted the initial papillary stone, because of the existence of urinary pH values below 5.5. It should be noted that COM crystals are very effective heterogeneous nucleants of uric acid crystals [26]. These stones should not be confused with the typical mixed uric acid/COM stones that are generated in cavities with low urodynamic efficiency, in which there is no contact of the COM with the papilla.

Some papillary stones have acicular sodium urate crystals around the stone-papilla junction, and these crystals sometimes appear together with Randall’s plaque (Fig. 5). This indicates that the formation of sodium urate after the formation of the intrapapillary Randall’s plaque is a consequence of urine with high pH and hyperuricosuria. Curiously, we also observed bacterial imprints the hydroxyapatite deposits in this area (Fig. 5H). This could indicate that the lesion on the surface of the papilla did not originate within the papilla, but instead was due to lesions generated by bacteria. In this case, the formation of urate crystals is a consequence of a high urinary pH and hyperuricosuria.

Finally, we detected small papillary COM stones in the absence of Randall’s plaque and other deposits that explain the development of COM crystals (Type V stones). In this case, columnar COM crystals appeared to begin development directly in the concave area of the stone, while in close contact with the papillary tissue (Fig. 6). The formation of this type of COM papillary calculus is more difficult to explain. The complete absence of hydroxyapatite residues in the area of the stone-papilla junction (Fig. 6E and F) may indicate that the calculus was generated directly on a small area of damaged epithelium. Thus, a subepithelial deposit of hydroxyapatite or another factor may alter the cuboidal epithelium that covers the papilla.

## Conclusion

Renal papillary stones can form by different pathways, although all of them are associated with tissue lesions generated by oxidative stress from cytotoxic agents, by high concentrations of calcium and/or oxalate, or by another insult that damages the cuboidal epithelium which covers the papilla. Obviously, a competent immune system can help prevent the development of these stones by eliminating the intratissue hydroxyapatite deposits or promoting regeneration of the outer epithelium.

## Data Availability

No datasets were generated or analysed during the current study.
